# Change in Municipality-Level Health-Related Social Capital and Depressive Symptoms: Ecological and 5-Year Repeated Cross-Sectional Study from the JAGES

**DOI:** 10.3390/ijerph16112038

**Published:** 2019-06-08

**Authors:** Ryota Watanabe, Katsunori Kondo, Tami Saito, Taishi Tsuji, Takahiro Hayashi, Takaaki Ikeda, Tokunori Takeda

**Affiliations:** 1 Department of Rehabilitation, Tsushima City Hospital, Aichi 496-8537, Japan; 2 Graduate School of Medical and Pharmaceutical Sciences, Chiba University, Chiba 260-8670, Japan; 3 Department of Social Preventive Medical Sciences, Center for Preventive Medical Sciences, Chiba University, Chiba 260-8670, Japan; kkondo@chiba-u.jp (K.K.); tsuji.t@chiba-u.jp (T.T.); 4 Department of Gerontological Evaluation, Center for Gerontology and Social Science, National Center for Geriatrics and Gerontology, Aichi 474-8511, Japan; 5 Department of Social Science, National Center for Geriatrics and Gerontology, Aichi 474-8511, Japan; t-saito@ncgg.go.jp; 6 Department of Rehabilitation and Care, Seijoh University, Aichi 476-8588, Japan; hayashi-taka@seijoh-u.ac.jp (T.H.); takeda@seijoh-u.ac.jp (T.T.); 7 Department of Health Policy Science, Graduate School of Medical Science, Yamagata University, Yamagata 990-9585, Japan; t.ikeda0110@gmail.com; 8 Department of International and Community Oral Health, Tohoku University Graduate School of Dentistry, Miyagi 980-8575, Japan

**Keywords:** depression, older adults, population approach, municipality level, social capital, Japan Gerontological Evaluation Study

## Abstract

Prevalence of depressive symptoms is lower in communities with greater social capital (SC). However, it is unclear whether a prevalence of depressive symptoms will decrease in communities where SC has increased. We investigated the relationship between the changes in municipality-level SC and depressive symptoms by using 5-year repeated cross-sectional data from the Japan Gerontological Evaluation Study. In 2010 and 2016, self-reported questionnaires were mailed to functionally independent residents aged 65 years or older living in 44 municipalities; valid responses were received from 72,718 and 84,211 people in 2010 and 2016, respectively. All scores were aggregated at the municipality level. The dependent variable was the change in the prevalence of depressive symptoms that were diagnosed with a 15-item Geriatric Depression Scale. Independent variables were the score of change in health-related SC indicators, e.g., social participation, social cohesion, and reciprocity. A multiple regression analysis was employed. The average prevalence of depressive symptoms decreased from 28.6% in 2010 to 21.3% in 2016. The increases in the percentages of sports group participation (B, −0.356), and reciprocity scores (B, −0.597) were significantly associated with the decrease in the prevalence of depressive symptoms after adjusting for potential confounding variables. Our findings suggest that community SC might be an intervention for protecting depressive symptoms in municipalities.

## 1. Introduction

Depression is the most common mental disorder (CMD) worldwide. According to the World Health Organization [1], the estimated prevalence of depression is 4.4%. According to the literature, depression in older adults has been associated with poor quality of life [2], functional decline [3], dementia [4], and mortality [5]. Thus, preventing depression is a critical target. 

Risk factors of depression in older adults include low education, acceptance of risky lifestyle practices (e.g., smoking, risk drinking, physical inactivity, and obesity), and chronic disease [6,7]. Recent findings have indicated that mental health including depression in late life might be protective in relation to social capital (SC) [8,9,10,11].

One should realize that SC is an umbrella term meaning that resources are accessed by individuals as a consequence of their membership status in a network or group [12]. The conception of SC is often measured using civic engagement, individual and community social networks, and trust in the community [13], which can all be divided into structured, cognitive, bonding, and bridging [12]. Furthermore, SC has two levels: individual and group. Individual-level SC refers to resources that individuals can access through individual egocentric networks. Group-level SC is an asset of the entire network and benefits individuals included therein [12]. 

Within SC, health-related SC has been commonly used to examine associations with health-related outcomes, including depressive symptoms [14]. Earlier works from De Silva [8] and Ehsan and De Silva [9] have reported a strong inverse correlation between individual-level cognitive SC and CMD. At the community level, researchers have reported that CMD was low where community-level cognitive SC was high for seven cross-sectional studies [8,9]. 

Much of the literature on the community level has been cross-sectional, and the scarcity of community SC and CMD research has been noted [8,9]. Ecological research is appropriate to assess the impact on an entire population that profits from the direct effect of SC on individuals [15]. Although SC has been demonstrated to be a modifiable factor [16,17], research has not determined whether decreases in community-level depression are related to increases in community SC. 

The purpose of this study was to examine whether the prevalence of community-level depressive symptoms decreased in a community where health-related SC increased using an ecological design.

## 2. Methods

### 2.1. Data

We used repeated cross-sectional data derived from the Japan Gerontological Evaluation Study (JAGES). The JAGES is an ongoing cohort study investigating social and behavioral factors related to health decline, including mortality and onset of functional or cognitive impairment among individuals aged 65 years or older [18,19]. Our data were collected from two waves of the JAGES, namely, 2010-12 (2010) and 2016. The mean follow-up period was 5.3 years. 

Self-reported questionnaires were mailed to independent residents not eligible to receive public long-term care insurance benefits. The questionnaires were mailed to 31 municipalities in the 2010 wave and 39 municipalities in the 2016 wave, and the questionnaires were collected from 112,123 and 196,436 participants, corresponding to response rates of 66.3% and 70.2%, respectively. Data from 21 municipalities were obtained at both time points, in 2010 (*n* = 80,318) and 2016 (*n* = 100,868), and we excluded (i) missing information on sex, age, depressive symptoms, and basic activity of daily living (BADL; 2010: *n* = 6056, 2016: *n* = 11,321) and (ii) individuals whose BADL was not independent (2010: *n* = 1544, 2016: *n* = 5336). In addition, ordinance-designated cities in Japan have wards with functions similar to municipalities. Therefore, we split the two ordinance-designated cities into 25 wards. 

Consequently, we used data from 44 municipal units (*n*: 2010 = 72,718 (minimum: 352, maximum: 5915), 2016 = 84,211 (minimum: 418, maximum: 8721)). Random sampling methods were used in 34 municipal units, and complete enumeration survey methods were used in 10 municipalities. From the 34 municipalities where the sampling methods were conducted, we tracked the participants of 2010 in 2016 to create panel data sets, and the data were oversampled.

Ethical approval for the study was obtained from the Nihon Fukushi University Ethics Committee (application number: 10-05), National Center for Geriatrics and Gerontology (application number: No. 992-2), and Chiba University Ethics Committee (application number: No. 2493). 

### 2.2. Dependent Variables

For the dependent variables of this community-level ecological study, we calculated the difference between the prevalence of depressive symptoms by subtracting the depressive symptoms for 2010 from the depressive symptoms for 2016 for each municipal unit. We assessed depressive symptoms by using the Japanese short version of the Geriatric Depression Scale (GDS) (score range: 0–15; Cronbach’s alpha = 0.80) developed for self-administrative surveys [20,21]. We followed the literature [22,23,24]; thus, mild or severe depressive symptoms (GDS ≥ 5) were the cutoff scores previously validated as a screening instrument for major depressive disorder with 96% sensitivity and 95% specificity [22]. 

### 2.3. Independent Variables

For the independent variables, we measured health-related SC in the 2010 survey and the 2016 survey, and the difference score was calculated by subtracting the scores for 2010 from the scores for 2016 for each municipal unit. We defined SC as “features of social organization, such as trust, norms, and networks, that can improve the efficacy of society by facilitating coordinated actions” [25], and health-related SC has been a commonly used concept in epidemiological studies [14]. We selected health-related SC based on the indicators in Saito et al. [14], which examined the validity of community-level SC for Japanese people 65 years or older.

Regarding social participation, we assessed the frequency of participation in volunteer groups, sports groups, and hobby groups [14]. We created two intervals. The first interval was as follows: <1 day/month and ≥1 days/month [10,14]. The second interval was as follows: <1 day/week and ≥1 days/week [26]. Regarding the frequency of contact with friends and acquaintances, this was divided into two categories: <1 day/month and ≥1 days/month [27]. Regarding social support, we surveyed emotional and instrumental support (i.e., providing and receiving). Social support was assessed by asking the following question “Do you have someone who listens to your concerns and complaints?” Providing instrumental support to others was evaluated using the question “Do you have someone you take care of when she or he is sick in bed?” These social support responses were dichotomized into yes and no [14]. 

In addition, we used SC scores developed by Saito et al. [14]. According to Saito’s index, civic participation (five questions), social cohesion (three questions), and reciprocity (three questions) were calculated for each municipal unit. Civic participation refers to the level of residents’ participation in community organizations and activities. Civic participation was originally comprised of five questions. However, because information on attachment to the community was not available in the 2010 wave, we calculated the score of civic participation based on three questions. Social cohesion pertains to the cognitive aspects of interpersonal trust. Reciprocity represents community social support.

### 2.4. Covariates

We age-standardized the all variables by applying direct methods to the Japanese demographical statistics of 1985, which the Ministry of Health, Labor and Welfare have been using as a standard of statistics [28]. For the adjusted variables, we measured socioeconomic status in the 2010 survey and the 2016 survey, and the score for 2010 was subtracted from the score for 2010 across each municipal unit. Socioeconomic status included change in proportion of living alone, change in proportion of people with an equivalized household income greater than 2 million yen [29], change in proportion of having an education level greater than 10 years [29], and change in the proportion of the employed. 

### 2.5. Statistical Analysis

First, we compared the differences in the study variables between the two time points by using a paired-samples *t*-test. Second, we used multiple linear regression models to examine the association between change in health-related SC variables and change in depressive symptoms (GDS ≥ 5). The following two models were constructed. In the crude model, change in health-related SC and change in depressive symptoms were separately modeled, and Model 1 added the covariates of change for living alone, change in proportion with an education level (greater than 10 years), change in proportion of having income (greater than 2 million yen for equivalized household income), and change in employment compared with the crude model. Notably, we took advantage of the change in the two time points, which can control for time-invariant unobserved and observed confounding community characteristics [30]. Finally, in 34 municipal units out of 44 municipal units, we tracked the participants of 2010 in 2016 to create panel data sets. The data were oversampled and in 2016 for participants obtained in 2010, they show a survival bias that was one of the selection biases. For that reason, we conducted the comparison using an independent-samples *t*-test to assess whether there was any difference in the change over the 5 years in the group of the complete enumeration survey and the group comprised by some individuals in the oversampling survey. We added sex to the analysis of Model 1 in the multiple linear regression and further investigated the interaction with sex and each health-related SC. As a result, of the 14 items for health-related SC, only social cohesion showed a significant association with changes in municipal-unit-level depressive symptoms. For that reason, sex was analyzed collectively. All analyses were conducted using SPSS V. 25.0J (IBM Japan, Tokyo, Japan).

## 3. Results

Overall, the prevalence of depressive symptoms decreased in 2016 compared with 2010. Age-adjusted average prevalence of depressive symptoms decreased from 28.6% (standard deviation (SD): 3.4%) in 2010 to 21.3% (SD: 2.7%) in 2016.

Table 1 presents the age-adjusted comparison in 2010 and 2016 for each variable. Compared with 2010, in 2016, the percentages of respondents who were married, lived alone, had an educational history of more than 10 years, and were employed increased. Of the 14 health-related SC items, we observed an increase in nine items and a decrease in three items, and the association was significant.

From 2010 to 2016, the prevalence of depressive symptoms decreased and much of the health-related SC increased.

Table 2 shows the crude and adjusted model for the multiple regression of changes in health-related SC with changes in depressive symptoms. Among the 14 indicators of health-related SC, a significant association was observed for 10 indicators. For example, change in sports group (≤1 days/week: B, −0.356), change in frequency of contact with friends (≤1 days/month: B, −0.440), and change in reciprocity (B, −0.597) after adjusting to the change in socioeconomic status all predicted health-related SC.

An association was shown between increased health-related SC and decreases in the prevalence of depressive symptoms after adjustment for potential confounding variables.

Supplementary Table S1 shows health-related SC for a 5-year change based on the sampling methods used. In the municipal units where the complete enumeration survey was carried out and a group with some oversampling was observed, we confirmed whether the change in the value from 2010 to 2016 was different in the two groups. Of the variables used as the dependent variable and independent variables, only change in social cohesion showed a difference between the two groups. In the case of a change in social cohesion, we performed multiple regression analysis and added the sampling methods’ difference as dummy variables to adjust for the variables. However, the results did not change. 

## 4. Discussion

According to our review of the literature, this is the first study to use a repeated cross-sectional design to verify that increases in the municipal unit level of health-related SC, such as civic participation, social cohesion, and reciprocity, are related to decreases in the prevalence of depressive symptoms in the municipal unit level. 

Our results demonstrate that the prevalence of depressive symptoms after 5 years decreased by an average of 7.3% points. Another study reported a secular trend in the average score of GDS using the complete enumeration survey in the same district of older adults. According to the results, the average score gradually decreased for men and women from 2003 to 2012 (GDS score annual average decrease value: men—0.001, women—0.09) and decreased significantly in women, and the reason for improvement was the influence of disability prevention project [31]. Furthermore, depression is a risk factor for suicide [7], and the suicide rate declined from 2010 to 2016 in Japanese older adults [32]. Therefore, there is a strong possibility that the prevalence of depressive symptoms is decreasing nationwide in Japan. The results of this study support these reports.

The health-related SC increased with many indicators from 2010 to 2016. According to previous studies, a secular trend is found for the index of Social Interaction using the complete enumeration survey in the same district of older adults [33]. According to that study, even though the interest in health-related SC increased over the last 20 years, SC in the form of social participation and social support did not change over those 20 years. One reason for the increase from 2010 to 2016 in this study could be the influence of Japanese disability prevention projects. 

Since 2015, the disability prevention project in Japan has undergone a major policy change from a high-risk approach to a population strategy [34]. Disability prevention activities focusing on participation in activities are being promoted as one of the action policies. According to the records of the Ministry of Health, Labor and Welfare [35], places where older people can easily be active and participate increased from 43,154 in 2013 to 76,492 in 2016, which is, 1.77 times in 3 years. Notably, the number of people participating has also increased, from 840,718 in 2013 to 1,439,910 in 2016, which is, 1.71 times in 3 years. However, these numbers likely underestimate the actual numbers because they are merely the numbers captured by the municipality, not the total numbers. In addition, older people participate in activities such as sports and hobby groups in private clubs, but municipal units are unaware of the participants’ presence. Thus, the number of participants is increasing even in places where the municipality does not comprehend; hence, the number of participants that actually exist is expected to be higher. 

The JAGES provided evidence for this policy change [19], and the collaborating municipal units are eager to consider this effort. The increase in the number of places where older people can easily be active and participate will lead to an increase in older people who engage in activities and participation, and this increase in people’s connections may have led to the increase in health-related SC from 2010 to 2016. 

By considering the association between SC type and change in depressive symptoms, we showed an association between the decrease in depressive symptoms for increases in structural SC and cognitive SC. Regarding the structural SC, social participation and frequency of contact with friends were extracted from this factor. In the systematic review, the structural SC of the area was not related to CMD [9], whereas a structural SC, such as a sports group, a hobby group, and civic participation, showed a decrease in depressive symptoms in the current study. 

In another study of JAGES, structural SC was divided into vertical and horizontal categories based on the result of factor analysis [36]. In vertical organizations, authority and resources are related hierarchically, whereas these two concepts are related equally in horizontal organizations. In relation to depression, a study demonstrated that the prevalence of depressive symptoms was low in areas where a high percentage of horizontal organizations, such as sports groups, adjusted individuals’ participation in a cross-sectional manner [10]. In longitudinal studies, older people living in districts rich in horizontal organization have reported few depressive onsets 3 years later [11].

In this study, structural SC may be related to the decrease in depressive symptoms because it deals with the horizontal structure through the structural SC. Social networks, such as the frequency of contact with friends, are known to affect health through psychosocial mechanisms. Regarding connection and mental health, happiness as a network phenomenon that extends up to three degrees of separation was reported (e.g., to the friends of one’s friends’ friends) [37].

For cognitive SC, an association was observed between an increase in social cohesion and reciprocity and a decrease in the prevalence of depressive symptoms. Another study reported that community-level cognitive SC and CMD are inversely associated [9]. By contrast, longitudinal research suggested that social cohesion and reciprocity at the community level were not associated with the onset of depression after 3 years [11]. As this study verified the association between change in health-related SC and change in depressive symptoms, the differences in the study designs might explain the different results. Further research is necessary.

These known mechanisms for community-level SC, namely (1) social contagion, (2) informal social control, and (3) collective efficacy, are the mechanisms that affect individual health [12]. In addition to those mechanisms, people’s connections may have formed a network of social support and reduced the prevalence of depressive symptoms. Regarding the relationship between people’s connections and depressive symptoms, a low prevalence of depressive symptoms in a community with high social support was reported [38]. A meta-analysis suggested that nonprofessional support is effective for reducing depressive symptoms [39], and it supported the association of community social support with depression. Due to the increase in municipal units' level of health-related SC, the prevalence of municipal units’ level of depressive symptoms may decrease through the aforementioned route.

## 5. Strengths and Limitations

This research has two strengths. The first strength is that the prevalence of depressive symptoms decreased, whereas health-related SC increased. This result was based on a longitudinal study using a large population with a wide range of urbanites and 150,000 people in 44 municipal units with various characteristics. The second strength was that we verified the validity of a core indicator of an age-friendly city. 

The results of this research may contribute to core indicators of socioenvironmental factors of an age-friendly city, which engages itself in volunteer and sociocultural activities recommended by the World Health Organization [40]. In Japan, after 2015, disability prevention focused on community development. This critical study suggests that increases in health-related SC, due to community development, may lead to decreases community-level depressive symptoms.

This research has three limitations. First, the target area of this study was 44 municipal units, which means that we could not verify the independence of each health-related SC, and there is a possibility that many factors could not be fully adjusted. However, we took advantage of the change between two time points, which may have controlled for time-invariant unobserved and observed confounding community characteristics [30].

Second, the use of repeated cross-sectional studies meant that we could not deny the possibility of reverse causality, such that increases in SC could have occurred as a result of decreases in depression. Other studies have demonstrated that the intervention promoting social participation in a community center increase health-related SC [16,17]. In Japan, the shift in the disability prevention measures allows the possibility that social participation in older adults is promoted by the increase in the number of places where older people can easily be active and participate, which can increase health-related SC.

## 6. Conclusions

We indicated that increases in municipality-level health-related SC factors, such as civic participation, social cohesion, and reciprocity, were related to decreases in municipality-level depressive symptoms using a 5-year repeated cross-sectional design, adjusting covariates. Our findings suggest that community SC interventions may reduce depressive symptoms in municipal units.

## Figures and Tables

**Table 1:** Age-adjusted descriptive statistics of community factors (municipal units = 44).

Variables	2010				2016					
	Minimum	Maximum	Mean	SD	Minimum	Maximum	Mean	SD	Difference (2016–2010)	*p* Values
% Depressive symptoms ^a^	23.2	39.5	28.6	3.4	16.3	27.9	21.3	2.7	−7.3	<0.001
% Married	60.1	78.3	71.5	4.9	60.2	80.2	72.5	4.8	0.9	0.003
% Living alone	8.0	27.1	14.9	4.9	9.2	31.3	17.0	5.2	2.1	<0.001
% Education ≥10 years	32.4	81.0	59.3	14.2	41.8	84.4	69.5	11.4	10.2	<0.001
% Equivalent income ≥2,000,000 JPY	34.2	66.3	52.2	8.9	37.1	63.8	51.6	7.4	−0.5	0.185
% Employed	15.9	35.9	23.7	4.1	22.7	46.1	31.0	4.6	7.4	<0.001
% Volunteer group ^b^ (≥1 per month)	7.7	15.4	11.4	1.7	10.9	20.2	15.7	1.8	4.3	<0.001
% Sports group ^b^ (≥1 per month)	11.4	31.6	24.2	4.3	19.9	39.4	30.7	4.6	6.4	<0.001
% Hobby group ^b^ (≥1 per month)	26.6	48.6	39.0	5.3	27.5	45.5	38.9	4.8	−0.1	0.869
% Volunteer group ^b^ (≥1 per week)	3.0	8.8	5.5	1.4	4.7	10.5	7.6	1.5	2.1	<0.001
% Sports group ^b^ (≥1 per week)	8.6	26.3	19.3	3.5	12.7	31.5	23.9	3.8	4.6	<0.001
% Hobby group ^b^ (≥1 per week)	15.4	32.2	24.5	3.8	16.3	29.5	24.2	3.3	−0.3	0.394
% Frequency of contact with friends ^b^ (≥1 per month)	67.3	82.5	73.4	3.6	66.5	81.6	72.2	3.8	−1.2	0.002
% Receiving emotional social support	89.3	96.4	93.7	1.4	92.1	97.0	94.7	1.2	1.1	<0.001
% Providing emotional social support	88.6	94.9	92.6	1.4	90.4	96.6	93.9	1.3	1.3	<0.001
% Receiving instrumental social support	86.6	97.0	94.0	2.4	87.4	97.4	94.7	2.1	0.7	<0.001
% Providing instrumental social support	81.0	90.7	87.2	2.2	81.0	90.4	85.5	2.1	−1.7	<0.001
Civic participation	41.1	75.0	61.3	8.3	47.2	84.5	69.0	8.4	7.7	<0.001
Social cohesion	143.3	182.7	161.0	10.1	138.2	172.9	158.7	9.0	−2.3	<0.001
Reciprocity	185.6	201.1	196.1	3.2	189.7	203.7	198.3	2.9	2.2	<0.001

SD: Standard deviation. * All factors were adjusted for age using the direct methods. ^a^ Depressive symptoms were defined as prevalence of Geriatrics Depression Scale ≥5 points. ^b^ Percentage of respondents that participated in the group.

**Table 2:** Multiple linear regression of changes in a health-related social capital (SC) with changes in depressive symptoms ^b^ (*n* = 44).

Variables	Crude			Model 1 ^d^		
	B	SE	*p* Values	B	SE	*p* Values
% Change in ^a^ volunteer group (≥1 per month)	−0.273	0.184	0.145	−0.203	0.168	0.236
% Change in ^a^ sports group (≥1 per month)	−0.270	0.123	0.033	−0.234	0.124	0.066
% Change in ^a^ hobby group (≥1 per month)	−0.341	0.103	0.002	−0.309	0.111	0.008
% Change in ^a^ volunteer group (≥1 per week)	−0.089	0.230	0.700	0.051	0.224	0.822
% Change in ^a^ sports group (≥1 per week)	−0.397	0.152	0.013	−0.356	0.157	0.030
% Change in ^a^ hobby group (≥1 per week)	−0.215	0.134	0.115	−0.124	0.134	0.361
% Change in ^a^ frequency of contact with friends (≥1 per month)	−0.507	0.108	<0.001	−0.440	0.124	0.001
% Change in ^a^ receiving emotional social support	−0.797	0.276	0.006	−0.688	0.271	0.015
% Change in ^a^ providing emotional social support	−0.992	0.168	<0.001	−0.938	0.163	<0.001
% Change in ^a^ receiving instrumental social support	−0.229	0.321	0.479	−0.765	0.331	0.026
% Change in ^a^ providing instrumental social support	−0.451	0.227	0.054	−0.676	0.196	0.001
Change in ^c^ civic participation	−0.218	0.065	0.002	−0.189	0.068	0.008
Change in ^c^ social cohesion	−0.081	0.083	0.333	−0.171	0.074	0.027
Change in ^c^ reciprocity	−0.562	0.135	<0.001	−0.597	0.127	<0.001
% Change in ^a^ living alone	0.041	0.231	0.859			
% Change in ^a^ educational attainment	−0.252	0.071	0.001			
% Change in ^a^ equivalent income ≥2,000,000 JPY	−0.154	0.120	0.207			
% Change in ^a^ employed	−0.116	0.140	0.414			

All factors were adjusted for age using the direct methods. ^a^ Percentage of 2016 subtracted by 2010. ^b^ Depressive symptoms were defined as prevalence of Geriatrics Depression Scale ≥5 points. ^c^ The value of 2016 subtracted by 2010. ^d^ Model 1 was adjusted for change in living alone, change in educational attainment (≥10), change in equivalent income (≥2,000,000 JPY) and change in employed.

## Supplementary Materials

The following are available online at https://www.mdpi.com/1660-4601/16/11/2038/s1, Table S1: Health-related social capital (SC) 5-year change by using sampling methods.
