# Supplementary material for: Change in Municipality-Level Health-Related Social Capital and Depressive Symptoms: Ecological and 5-Year Repeated Cross-Sectional Study from the JAGES
Source: Int J Environ Res Public Health. 2019 Jun 8;16(11):2038. doi: 10.3390/ijerph16112038 (PMC6604020; doi:10.3390/ijerph16112038)
Supplement: Supplementary file 1 [file ijerph-16-02038-s001.pdf]

International Journal of
Environmental Research and Public Health

MDPI

Article

Change in Municipality-Level Health-Related Social Capital and Depressive Symptoms: Ecological and 5-Year Repeated Cross-Sectional Study from the JAGES

Ryota Watanabe, Katsunori Kondo, Tami Saito, Taishi Tsuji, Takahiro Hayashi, Takaaki Ikeda and Tokunori Takeda

Table S1. Health-related social capital (SC) 5-year change by using sampling methods.

	Complete enumeration survey (*n* = 10)				random sampling (*n* = 34)				*p* value
	Minimum	Maximum	Mean	SD	Minimum	Maximum	Mean	SD	
% Change in [b] depressive symptoms [a]	−9.7	−5.1	−7.7	1.4	−11.9	−2.7	−7.2	2.2	0.496
% Change in [b] volunteer group (≥1 per month)	2.0	8.0	4.4	1.9	0.5	7.4	4.3	1.7	0.845
% Change in [b] sports group (≥1 per month)	2.8	10.1	6.2	2.1	−1.0	10.5	6.5	2.6	0.711
% Change in [b] hobby group (≥1 per month)	−1.4	3.3	0.5	1.4	−7.2	5.8	−0.2	3.1	0.445
% Change in [b] volunteer group (≥1 per week)	0.5	5.9	2.1	1.6	−0.9	5.7	2.1	1.4	0.909
% Change in [b] sports group (≥1 per week)	1.1	5.7	4.3	1.4	−0.8	8.0	4.7	2.1	0.572
% Change in [b] hobby group (≥1 per week)	−2.3	3.0	0.2	1.8	−6.9	4.0	−0.5	2.5	0.415
% Change in [b] frequency of meeting friends (≥1 per month)	−2.8	2.9	−1.0	1.6	−6.6	3.2	−1.3	2.6	0.779
% Change in [b] receiving emotional social support	0.1	2.2	1.2	0.6	−1.6	3.3	1.0	1.2	0.647
% Change in [b] providing emotional social support	0.2	3.0	1.3	0.8	−2.1	4.1	1.3	1.6	0.969
% Change in [b] receiving instrumental social support	−1.1	1.2	0.4	0.7	−1.4	3.7	0.9	1.1	0.168
% Change in [b] providing instrumental social support	−3.9	−1.0	−2.3	0.9	−6.2	1.0	−1.6	1.4	0.167

Change in [c] civic participation	6.3	17.1	11.1	3.9	−6.3	20.8	10.5	5.8	0.754
Change in [c] social cohesion	−10.8	−0.7	−6.4	2.9	−13.3	6.3	−2.0	4.8	0.015
Change in [c] reciprocity	0.4	6.1	2.9	1.5	−3.4	10.0	3.2	3.1	0.847

All factors were adjusted for age using direct methods

SD: Standard deviation

[a] Depressive symptoms were defined as the prevalence of Geriatrics Depression Scale ≥5 points

[b] Percentage of 2016 subtracted from 2010

[c] Value percentage of 2016 subtracted from 2010

© 2019 by the authors. Licensee MDPI, Basel, Switzerland. This article is an open access article distributed under the terms and conditions of the Creative Commons Attribution (CC BY) license (http://creativecommons.org/licenses/by/4.0/).
